# Imaging and Quantitation Techniques for Tracking Cargo along Endosome-to-Golgi Transport Pathways

**DOI:** 10.3390/cells2010105

**Published:** 2013-02-22

**Authors:** Pei Zhi Cheryl Chia, Paul A. Gleeson

**Affiliations:** The Department of Biochemistry and Molecular Biology and Bio21 Molecular Science and Biotechnology Institute, The University of Melbourne, Victoria 3010, Australia; E-Mail: p.chia@student.unimelb.edu.au

**Keywords:** optical imaging, super-resolution microscopy, flow cytometry, Golgi apparatus, endosomes, fluorescent proteins

## Abstract

Recent improvements in the resolution of light microscopy, coupled with the development of a range of fluorescent-based probes, have provided new approaches to dissecting membrane domains and the regulation of membrane trafficking. Here, we review these advances, as well as highlight developments in quantitative image analysis and novel unbiased analytical approaches to quantitate protein localization. The application of these approaches to endosomal sorting and endosome-to-Golgi transport is discussed.

## 1. Introduction: Intracellular Membrane Trafficking

The cell is the most basic functional unit of life. It has its roots in the Latin word *cellula*, which means “small room”. Ironically, the processes that ensure homeostasis in a cell are no small feat. The eukaryotic cell houses a number of membrane-bound compartments in which specific metabolic processes take place. The compartmentalization of eukaryotic cells confers considerable functional advantages, but also requires highly-tuned mechanisms to ensure that proteins are targeted to the appropriate compartment. One of the major processes responsible for the correct localization of molecules within the cell is membrane trafficking. In this process, membranous carriers bud off from a donor compartment and fuse with a recipient one, delivering their contents to a target organelle, whilst maintaining the fidelity of the donor compartment. Two major routes of membrane trafficking account for the intracellular targeting of proteins. The secretory pathway is responsible for the transport of nascent proteins from the endoplasmic reticulum to the plasma membrane and other endosomal organelles. The endocytic pathway is important for the uptake of proteins and substrates from the extracellular milieu and refers to transport from the plasma membrane to the endosomes.

**Figure 1:**
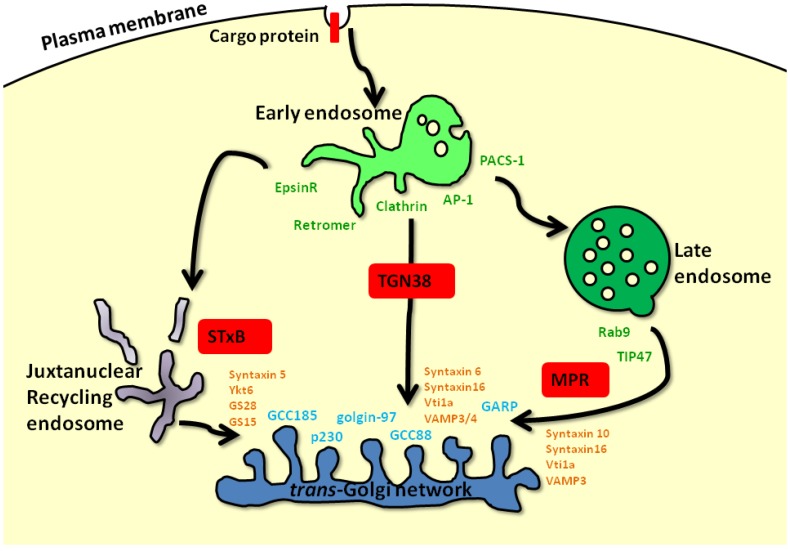
Machinery and cargoes along retrograde transport pathways.After internalization from the plasma membrane (PM) to the early endosome, protein cargoes (red rectangle) can undergo retrograde transport back to the TGN via a number of endosomal compartments. Shiga toxin B (STxB) and TGN38 utilize the early/recycling endosome-to-TGN route, whereas mannose 6-phosphate receptors (MPRs) use the late endosome-to-TGN pathway. Retrograde transport from the various endosomal compartments is mediated by different sets of machinery. The adaptor proteins, AP-1 and epsinR, aid in the recruitment of the clathrin and retromer coats to the early endosome, whereas late endosome-derived vesicles are characterized by the non-clathrin coat protein, TIP47. The processes of tethering, docking and fusion of transport intermediates with the *trans*-Golgi network (TGN) are mediated by distinct sets of machinery. Members of the TGN golgin family, p230, golgin-97, GCC88 and GCC185, and the multisubunit tethering complex, GARP, function as tethers (blue text). Distinct SNARE complexes mediate fusion events of different transport intermediates with the TGN (orange text). Reviewed in Bonifacino and Rojas, 2006, and Johannes and Popoff, 2008.

The early endosome is defined as the first endocytic compartment to accept incoming cargoes internalized from the plasma membrane (PM) [1]. The fate of an internalized protein at the early endosome can be any of the following: recycling back to the PM either directly or via the recycling endosomes, degradation at the lysosomes through retention of cargoes in the early/late endosomes or escape from the degradative pathway via transport from endosomes to the *trans*-Golgi network (TGN). Transport from the endosomes to the TGN can occur at multiple points along the endo-lysosomal network, and these distinct routes are collectively known as retrograde transport pathways [2,3] (Figure 1). Retrograde transport is utilized by a subset of proteins that undergo continuous cycling between the PM and intracellular endosomes. These so-called recycling proteins include sorting receptors, such as the mannose 6-phophate receptors (MPRs), sortilin and Wntless, transmembrane proteins, such as TGN38 and TGN46, the endopeptidase furin, the SNAREs, as well as ion and glucose transporters [2,3,4,5,6,7]. Retrograde pathways have also been hijacked by pathogens and toxins for entry into the cell. These include bacterial and plant toxins, such as Shiga toxin, Cholera toxin, pertussis toxin and ricin [6,8,9]. An increasing number of cargoes have been found to undergo retrograde trafficking, and these endogenous and exogenous cargoes have been instrumental in delineating retrograde transport routes and highlighting the plethora of transport machinery required for each process (Figure 1).

Recently, there have been major advances in improvements in the resolution of light microscopy and the development of new fluorescent probes, which in conjugation have a wide application in both cell and developmental biology. Of particular relevance to the topic discussed in this review and intrinsic to the analysis of retrograde transport pathways is the extensive use of fluorescent proteins to visualize cargoes and the ability to resolve dynamic membrane events at high resolution. This review will highlight recent innovations in fluorescent macromolecules, as well as advances in the imaging and quantitation, which are particularly pertinent to the study of membrane transport systems.

## 2. Super-Resolution Light Microscopy: Resolving Nanomolecular Events

Resolution is the minimum distance at which two objects can be distinguished when imaged by microscopy. The information we can discern from an image is intrinsically linked to its resolution. Diffraction of light is to resolution what Mr. Hyde was to Dr. Jekyll. Diffraction causes sharp points to appear blurry and is the basis of the Abbe's limit of resolution [10], which is about 200 nm laterally (*xy* plane) and 500 nm axially (*yz* plane). Conventional wide-field and confocal laser scanning microscopes are bound by this limit. Electron microscopy (EM) remains the gold standard for resolving objects in biological samples. However, EM lacks many of the advantages that fluorescence microscopy offers, such as the ability to perform life cell imaging and multi-colour labelling. Super-resolution microscopy refers to a suite of techniques that breach the diffraction barrier of light and provides enhanced resolution compared with traditional microscopic approaches. There are three major super-resolution techniques available, namely Structured Illumination Microscopy (SIM), Stimulated Emission Depletion Microscopy (STED) and fluorescent probe-based technologies, referred to as single-molecule Pointillism microscopy, which includes photoactivated localization microscopy (PALM) and stochastic optical reconstruction microscopy (STORM). Each of these techniques uses a different strategy to bypass Abbe's limit to allow sub-100 nm resolution; however, each has its individual strengths and weaknesses. The following will give a brief description of each of these super-resolution techniques. For a more detailed description and principles underlying these techniques, readers should source the following excellent reviews on this topic [11,12,13,14].

Structured Illumination Microscopy (SIM) centres on the often undesired artefact of digital images, called moiré patterns [15]. This is an interference pattern created by overlaying two grids with differing angles or mesh sizes. By overlapping a known consistent pattern over another unknown pattern, moiré fringes are created, providing information about the unknown pattern previously obscured by diffraction. The higher the spatial frequency of the known pattern, the better the spatial resolution. Non-linear, or Saturated, Structured Illumination Microscopy (SSIM) offers further improvements in resolution over linear SIM (whose spatial frequencies are also limited by diffraction), providing resolution of <50 nm [16]. SIM requires the application of a known pattern on a specimen, by illuminating the stripe pattern on the sample. Thus, the trade-off for increased spatial resolution is the loss of temporal resolution. This means that SIM is better for fixed samples as opposed to life cell imaging, due to the detrimental effect of prolonged exposure of samples. Significant data processing is also required to put the final image together, using a Fourier-transformed-based analysis of raw data sets [15]. A major advantage with SIM is that it is compatible with most fluorophores commonly used in confocal or wide-field microscopy. This allows the ease of manipulation of samples and the option to carry out multi-colour imaging on a super-resolution platform. Multi-coloured 3D-SIM can also be achieved by illuminating the specimen with three beams of interfering light and observing the interference pattern along *x*, *y* and *z* axes [17]. SIM has been used to gain new insights into the organization features of the pigment granules of retinal pigment epithelial cells [18] and the membrane network and membrane-mediated pathways critical for the establishment of infection of erythrocytes by the malaria parasite, *Plasmodium falciparum* [19].

Stimulated Emission Depletion Microscopy (STED) is an extension of laser scanning confocal microscopy. In confocal microscopy, a focused beam of light is used to scan the specimen and the fluorescence signal from each spot is collected after passing through a spatial filter (generally a pinhole aperture). This point illumination effectively blocks signals from out-of-focus areas of the specimen. The size of the illuminated spot determines the resolution of the microscope; however, the light beam can never be focused more tightly than the diffraction limit, placing a ceiling on the resolution of conventional confocal microscopes. In STED, fluorophores are similarly excited by a focused laser beam. Before spontaneous emission of fluorescence occurs, a second doughnut-shaped laser beam illuminates the specimen and forces molecules within the doughnut to return to their ground state, by stimulating emission of a photon of the same wavelength. Thus, STED effectively switches off a subset of fluorophores, save for those in the centre of the doughnut. The resolution of STED can be improved by increasing the intensity of the doughnut-shaped STED beam, as this results in the sharpening of the remaining fluorescent spot to a size much smaller than the diffraction-limited focus beam. Typically, 30–80 nm resolution can be achieved [20,21,22], and less than even 10 nm resolution has been obtained [23].

While the doughnut-shaped depletion beam results in efficient depleting of fluorescence emission in the lateral direction, it does not enhance axial resolution. To do this, a third beam must be used to quench spontaneous fluorescence in the axial direction. This has been shown to be feasible, proving that STED is suitable for 3D super-resolution imaging [24,25]. Multicolour imaging in STED is particularly challenging, as two laser wavelengths are required per dye: one for excitation and one for depletion. This means that four laser beams of different wavelengths are needed for two-colour imaging, increasing the risk of interference. Nonetheless, this difficulty has been circumvented by sequential scanning of each colour [26,27] or choosing compatible fluorophores [20,28]. STED is also relatively fast for small fields of view and requires no postprocessing of data; however, photobleaching of fluorophores is a major concern due to high beam intensities.

Pointillism microscopy uses the ability to detect single fluorescent molecules to generate a super-resolution image, and in 2006, three independent groups each developed variations of this technique, which are named photoactivated localization microscopy (PALM) [29], fluorescence photoactivation localization microscopy (FPALM) [30] and stochastic optical reconstruction microscopy (STORM) [31]. These techniques share the principle of stochastically switching on individual fluorescent molecules spaced further apart than the diffraction limit so that an array of well-separated magnified diffraction spots can be recorded on a highly sensitive camera. The main difference between PALM and STORM is the type of fluorophores used. Photoactivatable fluorophores are fluorescent proteins that can be activated to initiate fluorescence emission from a quiescent state, while photoconvertible fluorescent proteins can be optically converted from one emission bandwidth to another. In PALM, photoconvertible fluorescent proteins are used, whose emission wavelength can be optically converted from one wavelength to another. This was first carried out with the photoconvertible Eos Fluorescent Protein (EosFP) [29]. EosFP normally emits green fluorescence at 516 nm, but emits at 581 nm instead upon irradiation at 400 nm. Only a small subset of proteins is photo-converted at a time for imaging in the yellow region (581 nm). Once this subset is photobleached, the process can be repeated for another subset of EosFP, until the entire population of EosFPs are photoconverted. Calculation of the exact coordinates of the single fluorescent molecules within the photoactivated population enables precise localization. Thus, the image is assembled one molecule at a time by means of iterative switching cycles. The reconstructed images feature optical resolutions of down to 10 nm. This process also forms the basis of Fluorescence Photoactivated Localization Microscopy (FPALM), where photoactivatable GFP is used instead [30]. 3D PALM has been achieved by combining PALM with a microscopy detection scheme, which has an improved axial resolution (4Pi microscopy) in interferometric PALM (iPALM), providing sub-20 nm 3D resolution [32]. PALM has recently been used to dissect the spatio-temporal events associated with activation of the T-cell receptor and has uncovered the new finding that TCR engagement results in recruitment and phosphorylation of the signalling adaptor protein, Lat, from subsynaptic vesicles [33]. There is now considerable potential to extend this technique to many other membrane trafficking events. One benefit of using PALM is the ability to carry out high-resolution imaging without complex hardware. However, it relies heavily on data postprocessing and is relatively slow in image acquisition. A derivative of PALM, PALM with independent running acquisition (PALMIRA) offers improvements on the speed front [34]. PALMIRA combines the previously separate steps of activation and readout, thus accelerating the process of acquisition.

STORM, on the other hand, uses photoswitching fluorescent dyes, such as Cy5 or Alexa647 [31,35]. STORM was first introduced using Cy3-Cy5 dye pairs, where Cy5 functions as the primary fluorophore, and can be switched between a fluorescent or a dark state in a reversible manner by 532 nm or 633 nm light, respectively. Recovery from the dark state to the fluorescent state depends critically on the proximity of the second dye Cy3 [35]. This Cy5-Cy3 “switch” can be cycled on and off thousands of times before becoming permanently photobleached. More recently, direct STORM (dSTORM) was introduced, showing that similar photo-switching properties can be achieved in several fluorescent dyes without the use of secondary reactivator fluorescent dye labels like Cy3 [36]. 3D-STORM has been achieved by introducing astigmatism into the optical path of the image with a cylindrical lens, which causes fluorophores to appear elliptical [37]. By fitting the image with a 2D elliptical Gaussian function, the *z* position of the fluorophore can be determined to high precision using its ellipticity.

## 3. Correlative Light and Electron Microscopy

An alternative and powerful approach to obtain high resolution of the cellular structures associated with fluorescent probes is the integration of both light and electron microscopy, referred to as correlative light and electron microscopy (CLEM). While fluorescent light microscopy can rapidly provide results on large sets of data, such techniques are generally limited in spatial resolution due to the wavelength of light. Transmission electron microscopy (TEM) on the other hand uses high-energy electrons (which have an extremely short wavelength) to achieve spatial resolution down to 1–3 nm. The integration of the two techniques allows for a deep insight into the biological processes that is not possible from the individual technique alone [38]. Although a potential limitation of this technique is the inability to observe dynamic processes in real-time, live-cell and/or confocal laser microscopy can be combined with TEM in CLEM.

CLEM provides the ability to locate a fluorescently labelled protein, such as a cargo protein within microdomains of organellar membranes and within transport carriers, to provide better understanding of the molecular details of sorting and trafficking events between the early endosomes and the Golgi [39]. One very effective strategy is to combine video imaging of live cells with immuno-electron microscopy. The use of GFP-tagged chimeras allows the behaviour of intracellular structures in a live cell to be followed, which can then be fixed at the moment of interest, for subsequent immuno-election microscopy analysis. The process can be described as taking a high-resolution snapshot of an interesting live event. One of the first examples of this technique was reported by Polishchuk *et al*. [40] in a study, which demonstrated that transport carriers operating between the Golgi complex and the plasma membrane differ from the expected small round vesicles. More recently, CLEM has also been used to characterize the 3D ultrastructure of the endo-lysosomal system [41] and the kinetics and localization of LAMP1 [42], as well as membrane trafficking between organelles [43].

## 4. Recent Innovations in Fluorescent Proteins

The resolution limit of the various super-resolution techniques is intrinsically tied in with the properties of available fluorophores. Thus, the optimization of the photophysical properties of fluorophores is of significant importance for advancing imaging approaches. Since the discovery of green fluorescent protein (GFP) from the luminescent jellyfish *Aequorea Victoria*, substantial improvements have been made in terms of brightness, photostability and colour palette [44,45,46,47]. However, the ability to convert GFP to variants emitting in the orange and red spectral regions remained a challenge until the surprising discovery of naturally occurring red fluorescent protein [48]. One such red fluorescent protein was identified in the sea anemone *Discosoma sp*. by Tsien and colleagues and was named DsRed [49]. Siebert and colleagues were able to generate a surprising mutant of DsRed (DsRed-E5) that was able to change fluorescence over time: from an initial bright green fluorescence to yellow orange and, finally, to red [50]. The ability to change its emission wavelength over time meant that DsRed-E5 could be used as a “Fluorescent Timer (FT)” and could be used to visualize spatiotemporal molecular events. Unfortunately, these red fluorescent proteins (RFPs) were obligately tetrameric, limiting their application to protein tagging and were often toxic or disruptive [48,49].

An improved monomeric version of DsRed was later made through multiple mutations [51,52,53]. Using one of the monomeric variants of DsRed, mCherry as a springboard, Verkhusha and colleagues developed a series of monomeric FTs that overcame this problem and could thus be fused to proteins-of-interest at the DNA level [54]. This new set of FTs consists of three mCherry-derived variants exhibiting distinct fast, medium and slow blue-to-red temperature-dependent chromophore transitions [54]. Importantly, FTs can be used to study trafficking of cellular proteins and to provide insight into the timing of intracellular processes. The sequence of events during trafficking of proteins before they reach their final compartment has often been subject to controversy. The use of FTs presents the potential to resolve some long-standing issues in trafficking itinerary of various proteins. As a proof of principle, Verkhusha and colleagues fused the Medium-FT to LAMP2A, to show that localization of LAMP2A to lysosomes is largely due to an indirect pathway from the PM to early/recycling endosomes before reaching the lysosomes [54].

The study of the complex photophysical properties of fluorescent protein variants has led to the generation of fluorophores that can undergo either photoactivation or photoconversion. Both photoactivatable and photoconvertible fluorescent proteins can be used in the controlled highlighting of distinct molecular pools within the cell and are ideal for investigation of protein dynamics in live-cell imaging [55,56,57]. These fluorophores offer a gentler alternative to the relatively harsh photobleaching techniques, such as fluorescence recover after photobleaching (FRAP) and fluorescence loss in photobleaching (FLIP).

The first photoactivatable (PA) optical highlight PA-GFP was a derivative of wtGFP, resulting from the substitution of histidine for threonine at position 203 (T203H), which is devoid of green fluorescence until activated [58]. Irradiation with intense violet light (390–415 nm) produces a 100-fold increase in green fluorescence (emission peak at 504 nm), allowing the tracking of molecular subpopulations. The PA-FP PAmCherry1 has been developed, which when used in conjunction with PA-GFP, allows simultaneous dual colour photoactivation imaging in live cells [54]. EosFP was originally used in PALM [29], but exists as a tetramer in solution, limiting its use in fusion proteins. More recently, mEos2 has been developed, which showed minimal oligomerization artefacts, while offering optimal brightness [59]. Developments have also been made in photoswitchable dyes. Initial super-resolution mapping applications were limited to Cy3-Cy5 dye pairs [60]. Closer examination revealed that photoswitchable fluorophores were more common than thought. Other cyanine dyes, such as Alexa647 [36] and ATTO655 [61], are also capable of photoswitching under reducing conditions [62].

Dronpa is a monomeric FP derived from *Pactiniidae* through directed and random mutagenesis [63,64] and belongs to a new generation of reversible optical highlighters with on-off switching capabilities. Dronpa exhibits unusual photochromic behaviour characterized by its ability to toggle fluorescence on and off following illumination with two different excitation wavelengths. Newer fluorescent proteins, such as mIrisFP, couple irreversible photoconversion from a green- to a red-emitting form with reversible photoswitching between a fluorescent and a nonfluorescent state in both forms [65]. Such proteins offer the potential to carry out PALM with pulse chase experiments for high resolution imaging of dynamic events.

The generation of hybrid probes that can be visualized by both light microscopy and EM is particularly important in CLEM. Most studies rely on the use of primary antibodies against GFP for analysis by immuno-EM. Grabenbauer and colleagues first demonstrated the photo-oxidation of 3,3'-diaminobenzidine (DAB) through GFP [66], named as the GFP recognition after bleaching (GRAB) technique. This involves GFP bleaching and photo-oxidation of the DAB into an electron-dense precipitate that can be visualized under EM. However, GFP possesses only sub-optimal photo-oxidation potential. A new fluorescent flavoprotein with good photo-oxidation properties, known as mini singlet-oxygen generator (miniSOG), was engineered from *Arabidopsis* phototropin 2 and can be fused to proteins instead of GFP [67]. However, miniSOG requires cofactors, such as flavin mononucleotide (FMN), to fluoresce, interfering with the cell's own FMN levels. Furthermore, miniSOG produces high levels of reactive oxygen species during illumination, potentially limiting its use in live cells. The search continues for tags that can produce high amounts of reactive oxygen species after fixation, but not *in vivo*.

## 5. Tools for the Analysis of Colocalization

The precise cellular location of a protein is critical in understanding its biological role. The colocalization of a protein-of-interest with well characterized markers by fluorescence microscopy has now become a routine approach in determining where it resides. However, colocalization analysis in optical microscopy is a process that can be ambiguous and inconsistent. Digitally, colocalization can be defined as the spatial overlap of two or more fluorophores. Physiologically, colocalization refers to the presence of two or more different molecules within the same cellular structure. These two definitions, although similar, do not equate to each other. Some studies have misinterpreted colocalization by assigning functional significance based on colocalization *per se* [68]. When carrying out colocalization analysis, one has to consider the dimensions of a cell. Cells are very crowded environments, and the separation of individual molecules requires imaging on the nanometre scale. This is far below the resolving ability of conventional microscopes, which is in the micrometre range. The accuracy of any claim concerning colocalization is bound by a number of factors: (1) the reliability of compartment markers, (2) the limits of the optical system and image acquisition (reviewed in super-resolution section) and (3) the mastery of image processing and analytical techniques. This section focuses on the techniques available for the quantitation of colocalization.

There are two main ways to evaluate colocalization events: (1) a global statistic approach that performs intensity correlation coefficient-based (ICCB) analyses and (2) an object-based approach. ICCB tools revolve around the use of statistics to assess the relationship between fluorescent intensities. This is usually carried out using correlation coefficients that measure the strength of the linear relationship between the intensity values of green and red pixels in a dual-colour image. The most common way of doing this is by determining the Pearson’s correlation coefficient. This is simply a scatter plot of the pixel grey values of two images against each other, where the intensity of pixels in the green image is the *x* axis and the intensity of pixels in the red image is the *y* axis. The slope would reflect the relative stoichiometry of both fluorophores (modulated by relative detection efficiencies). Pearson’s coefficient provides an estimate of how well the scatter fits to the slope. Its value can range from 1 to −1, with 1 representing complete positive correlation and −1 representing complete negative correlation. Pearson's coefficient points to colocalization especially for values close to 1. However, this analysis is particularly affected by instances where there is partial colocalization, noise and bleed-through and variations in fluorescence intensities; thus, in the mid-range (~0.5), colocalization conclusions should not be drawn from Pearson's coefficients [69].

Mander's coefficient is another commonly used measure of colocalization. In situations where the intensity of one fluorophore is a lot stronger than the other, Mander’s coefficient can be employed, as it removes average intensity values out of the mathematical expression [70]. This coefficient varies from 0 to 1, where 0 refers to non-overlapping images and 1 reflects 100% colocalization. The M1 coefficient is defined as the ratio of the “summed intensities of pixels from the green image for which the intensity on the red channel is above zero” to the “total intensity in the green channel”, and M2 is the converse for the red pixels. Thus, the Mander's coefficients are good indicators of the proportion of the green signal coincident with a signal in the red channel over its total intensity, even when there is a large difference in intensities. Other ICCB approaches are reviewed in detail in [69,71]. The public domain tool, named JaCoP (http://rsb.info.nih.gov/ij/plugins/track/jacop.html), groups together the most important ICCB tools, allowing the comparison of various methods.

A major drawback of all ICCB approaches is that spatial information is lost in determining the degree of colocalization. In contrast, object-based analysis involves the identification of “objects”, for example, endosomes, and subsequent measuring of colocalization within each of these structures. At its most primitive, this is done by performing a line scan: drawing a line across a structure and plotting the fluorescence intensities for two different channels across that line. Overlapping structures should result in matching fluorescence intensity profiles. However, this method is time consuming and is limited to larger and elongated structures, like Golgi stacks [72]. Object identification has now been automated using basic image segmentation tools, which identify objects based on a simple rule: pixels from true structures should have higher intensities than pixels from background noise. A threshold is first applied to an image, where pixels with intensities above a certain limit are considered part of an object. Edge detection is then carried out to delineate the object. This is usually achieved using filters that highlight the edges of structures based on sudden changes in intensity and is, in itself, subject to rigorous study [73]. Object-based colocalization can then be carried out in a number of ways. One method is to determine the centroids (geometrical centres) and intensity centres of objects and to compare the positions of centroids or intensity centres between two channels [74]. Objects are considered to colocalize if the distance between their centres is less than the resolution limit of the microscope [75].

A less restrictive measure of colocalization was developed [74], where two objects are considered to colocalize if the centroid of one object falls in the area covered by another object. This method is available as the plug in “3D object counter” (http://rsb.info.nih.gov/ij/plugins/track/objects.html), to process images and allow automated colocalization. Hamilton and colleagues describe another tool for measuring object based colocalization [76]. Organelle-Based Colocalization (OBCOL) was developed to measure colocalization in dynamic and multivariate structures, like recycling endosomes. This method automatically segments and quantitates colocalization across individual punctuate organelles and can be applied to 2D and 3D images. A number of colocalization statistics are then presented, such as number of objects, position and degree of overlap. OBCOL is an open-source ImageJ plug-in, available for download from http://obcol.imb.uq.edu.au/. The main drawback with OBCOL is that information on pixel intensity is lost along the way. Once images are thresholded, pixels are either given a value of “0” (for non-fluorescent) or “1” (fluorescent). Jaskolski and colleagues [77] proposed a method that instead combines object-based colocalization with ICCB analysis, providing a visualization of colocalization with correlation data. This method is particularly suitable for structures that vary considerably in size and shape, as well as fluorescence intensity. It is particular amenable for analysing the location of cargo in the endosomal-to-Golgi transport pathways.

## 6. High Throughput Quantitation of Intracellular Events by Pulse Width Analysis

**Figure 2:**
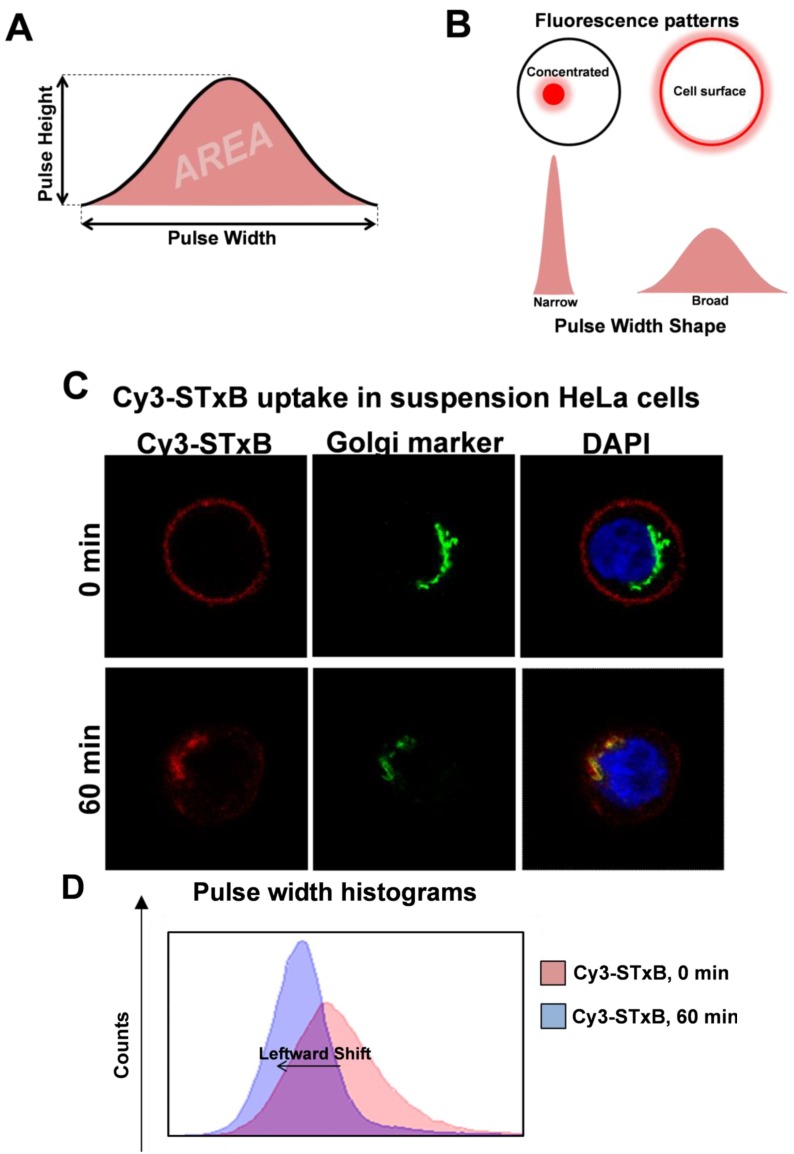
Flow cytometry pulse width can be used for quantitation of cargo localization**. (A)** The fluorescence signal of a fluorescent particle as it moves through the detector of a flow cytometer. The maximum height of the signal is the pulse height, and the pulse width is a measure of the return of the signal to a basal level. The total area under the curve is the pulse area. **(B)** The different pulse width shapes resulting from either a concentrated small area of intracellular fluorescence or cell surface fluorescence. **(C)** A Cy3-STxB internalization assay was carried out in HeLa cells in suspension. Briefly, a single cell suspension of HeLa cells was incubated with Cy3-STxB on ice and either fixed with 4% paraformaldehyde/PBS immediately (0 min) or allowed to internalize at 37°C for 60 min, prior to fixation (60 min). Cells were quenched with 50 mM NH_4_Cl/PBS, permeabilized with 0.1% Triton X-100/PBS and blocked with 5% FCS/PBS, followed by staining with mouse anti-GM130 (green) and DAPI (blue). A coverslip was applied onto a drop of the cell suspension on a glass slide and sealed with varnish for imaging on a Leica SP2 confocal microscope. **(D)** The pulse width histograms from flow cytometry analysis of 10,000 HeLa cells from each time-point of the Cy3-STxB internalization assay. At 0 min, the pulse width distribution is broad (pink), but this distribution becomes narrower and shifts to the left after 60 min of internalization at 37°C (blue).

Currently available quantitation techniques for looking at intracellular localization all have one thing in common: they require laborious and time-consuming image acquisition. Although the process of quantitating colocalization has become for the most part automated, images of at least 20–25 cells per condition need to be collected to produce significant data. Flow cytometry holds promise as high-throughput method for quantitation, as it has the ability to image thousands of particles per second. Pulse height, pulse area and pulse width are the most basic measurements given by a flow cytometer when a particle moves through a laser beam (Figure 2A). Pulse area is the most commonly plotted value in FACS analyses and is the total amount of light (e.g., fluorescence) emitted by a particle. Pulse height is the maximum value of the pulse, whereas pulse width provides information about the size of the particle [78]. Pulse width has been used in the past to discriminate a pulse due to a single cell from a pulse from a doublet (two cells stuck together) [79]. However, pulse width can be used for other purposes other than doublet discrimination. Hatters and colleagues recently developed a fast and simple method to analyze the degree of aggregation of Huntingtin in cells [80]. This technique relies on the measurement of pulse width and was termed Pulse Shape Analysis (PulSA). Using overexpression of GFP-tagged poly-glutamine repeats of different lengths, they showed that populations of cells with concentrated fluorescence (corresponding to aggregated fluorescent protein) have a narrower pulse width compared to cells with uniform cytosolic staining (non-aggregated protein).

Thus, different localized populations of fluorophores should theoretically result in different pulse widths (Figure 2B). We have used pulse width to track retrograde trafficking of the Cy3-conjugated non-toxic B subunit of Shiga toxin (Cy3-STxB) in HeLa cells [80]. Cy3-STxB binds to glycolipid Gb3 on membranes [81]. At the restrictive temperature of 4°C, Cy3-STxB cannot undergo internalization and remains at the cell surface (Figure 2C). The pulse width profile of surface-bound Cy3-STxB is a broad histogram (Figure 2D). Upon shifting to the endocytosis-permissive temperature of 37 °C, Cy3-STxB is rapidly internalized and undergoes retrograde transport. After 60 min at 37 °C, the Cy3-STxB is neatly localized to the Golgi. This shift in localization—from the cell surface to the Golgi—is reflected in the corresponding shift in pulse width histograms. The pulse width histogram for 60 min at 37 °C has shifted leftward compared to the 0 min time-point, indicating a decrease in pulse width concomitant with a more compact fluorescent signal in cells (Figure 2D).

Thus pulse width can be used to track the internalization and retrograde trafficking of cargoes in cells. This technique holds particular advantages over current methods for quantitating retrograde trafficking events. Under flow cytometry, cells with very low fluorescence signal, which may otherwise be ignored under fluorescence microscopy, can be detected. This allows for the use of lower expression rates of transfected constructs, which may be more physiologically relevant than the high expression levels needed for imaging under fluorescence microscopy. In addition, the ability to simultaneously detect up to eight colours opens up possibilities for tracking multiple processes in one sample. Flow cytometry also offers an obvious leap in the process of quantitation, which hitherto has been time-consuming and heavily reliant on complex algorithms. Although optimization is required to streamline the technique for sensitive quantitation of other trafficking processes, we envision that pulse width will be widely used in future to detect and quantitate shifts in localization or different trafficking routes.

## 7. Concluding Remarks

Multicellular organisms have a greater complexity in their retrograde transport pathways than single cell yeast, both in components and number of pathways. This is compounded by the heterogeneity within the early endosome, the compartment at which cargo sorting occurs after endocytosis from the plasma membrane. The pleomorphic appearance of the early endosomes can be attributed to the presence of regions of thin tubular extensions (~60 nm in diameter) and large vesicles (~400 nm) harbouring membrane invaginations [82]. These distinct juxtaposing subdomains mediate the numerous sorting events that occur at this compartment. Membrane tubules emanating from the endosomes can sort membrane proteins and lipids directly back to the PM. These tubules are also thought to result in the biogenesis of the endocytic recycling compartment. The main body of the maturing endosome eventually becomes a late endosome, carrying along luminal content and lipids and proteins associated with the early endosomal limiting membrane. The early endosome maintains links with the recycling endosome and late endosome, resulting in a high degree of cross-talk between compartments. The ability to localize proteins or follow their trafficking is entirely dependent on the integrity of markers for various compartments. The exclusivity of currently used markers for a particular compartment is under constant debate and is compounded by continuity of the endo-lysosomal system. These issues need to be resolved in order to reach firm conclusions on the localization and trafficking of various cargoes.

Three distinct retrograde pathways have so far been defined in mammalian cells. The evolution of these pathways is likely to reflect specialized functions associated with the various physiological processes of multicellular organisms. Current findings indicate a high degree of specificity in the choice of retrograde transport pathway for individual cargoes, implying the existence of transport-specific sorting signals [3,83]. However, the mechanisms for sorting at the early endosomes and the generation of transport intermediates for retrograde transport need to be better defined. The identification and detection of such transport intermediates will require the use of sophisticated imaging systems. High-resolution live imaging will continue to play an important role in identifying populations of cargo-loaded transport carriers emerging from the early and late endosomes. For example, transport intermediates from the early endosome to the TGN have been reported to arise from the tubular structures of the TSE/TEN [84]. By 3D electron tomography, these transport intermediates are usually non-branched, short tubules and vesicles without a clathrin coat, found in close proximity to the early endosome [84]. The application of CLEM holds considerable promise in further unravelling the events associated with endosomal sorting and trafficking. Notably, CLEM has recently revealed fine details of the dynamic processes associated protein machinery assembly, such as clathrin, to provide a 4D ultrastructural movie of the events associated with endocytosis [85].

Further application of super-resolution microscopy and new fluorophores will also continue to expand the applications of multiple colour imaging. Super-resolution methods, such as PALM and STORM, require a less complicated setup, compared to STED and SIM, and are thus more accessible. However, these two techniques require the use of photoactivatable/photoconvertible fluorophores. Photoconversion wavelengths are currently in the ultraviolet regions, which undermines their use in live imaging due to cell toxicity. It is expected that the engineering of optical highlighter proteins that shift photoconversion illumination wavelengths to the blue and green spectral regions (significantly less toxic to living cells) will greatly enhance the utility of PALM and STORM in nano-imaging. Another setback to super-resolution microscopy is photobleaching of fluorophores. Photobleaching limits the number of photons that can be detected from a single fluorophore and thus reduces the ability to localize that fluorophore. This limitation is compounded in live imaging where photobleaching is particularly deleterious to cells and would require fluorophores that are bright to begin with. Quantum dots have immerged as an ideal tool for use in super-resolution in terms of brightness and robustness against photobleaching. Quantum dots are semiconductor nanocrystals with very high extinction coefficients and superior photostability compared to fluorescent dyes and proteins [86]. Blinking is a phenomenon intrinsic to quantum dots [87], akin to the photoactivation of fluorophores during PALM or STORM. Quantum dots were used to image the cytoskeleton of CHO cells with a spatial resolution lower than 10 nm, using a total internal reflection fluorescence (TIRF) microscope [88]. More recently, multi-colour quantum dot complexes have been developed to allow for dual colour imaging [89]. Importantly, quantum dots have been proven for use in live cells, where transferrin-conjugated quantum dots were efficiently internalized by receptor-mediated endocytosis in HeLa cells [90], reflecting its potential for use in nano-imaging in live cells.

The development of unbiased quantitative methods for analysis of membrane transport and the behaviour of intracellular organelles remains a major challenge for cell biologists. The convergence of flow cytometry and imaging techniques holds promise for rapid collection of information on large numbers of cells, and removes the potential biasing from visual microscopic observation. Another key issue of relevance for defining intracellular membrane trafficking systems is the organization of the individual intracellular organelles and their topological relationship within the cell. A recent approach involves forcing cells into adopting a defined shape and subsequent analysis of the organization and 3D spatial relationships of different membrane structures, including early endosomes, the Golgi and Golgi-derived transport vesicles in a large number of cells [91]. Construction of density maps revealed a high degree of reproducibility in their steady state organization and also provided the capacity to detect subtle changes in intracellular membrane organization [91,92]. This approach holds considerable promise for the global assessment of the impact of membrane perturbation reagents on the spatial organization of intracellular membrane compartments.

In summary, the improvements in resolution of light microscopy, the integration of a number of different imaging systems, the further development of fluorescent probes, together with the application of powerful mathematical tools for analyses, will continue to provide major advances in understanding the regulation of membrane trafficking events and the associated dynamics of membrane organization at the molecular level. We are now well primed to undergo another giant leap in our understanding of molecular processes.
